# A prospective study to evaluate the accuracy of rapid diagnostic tests for diagnosis of human leptospirosis: Result from THAI-LEPTO AKI study

**DOI:** 10.1371/journal.pntd.0009159

**Published:** 2021-02-19

**Authors:** Janejira Dinhuzen, Umaporn Limothai, Sasipha Tachaboon, Panadda Krairojananan, Bangon Laosatiankit, Sakarin Boonprasong, Nuttha Lumlertgul, Sadudee Peerapornratana, Nattachai Srisawat

**Affiliations:** 1 Excellence Center for Critical Care Nephrology, King Chulalongkorn Memorial Hospital, Bangkok, Thailand; 2 Critical Care Nephrology Research Unit, Faculty of Medicine, Chulalongkorn University, Bangkok, Thailand; 3 Master of science program in Medical Science, Faculty of Medicine, Chulalongkorn university, Bangkok, Thailand; 4 Department of Entomology, Armed Forces Research Institute of Medical Sciences (AFRIMS), Bangkok, Thailand; 5 Sisaket Provincial Public Health Office, Sisaket, Thailand; 6 Division of Nephrology, Department of Medicine, Faculty of Medicine, Chulalongkorn University, and King Chulalongkorn Memorial Hospital, Bangkok, Thailand; 7 Department of Laboratory Medicine, Faculty of Medicine, Chulalongkorn University, Bangkok, Thailand; 8 Academy of Science, Royal Society of Thailand, Bangkok, Thailand; 9 Tropical Medicine Cluster, Chulalongkorn University, Bangkok, Thailand; 10 Excellence Center for Critical Care Medicine, King Chulalongkorn Memorial Hospital, Bangkok, Thailand; Rocky Mountain Laboratories, NIAID, NIH, UNITED STATES

## Abstract

**Background:**

Rapid diagnostic tests (RDTs) have become widely used in low-resource settings for leptospirosis diagnostic. This study aims to evaluate the diagnostic performance of the five commercially available RDTs to detect human IgM against Leptospira spp. in Thai population.

**Methodology/Principal findings:**

Ninety-nine serum samples from Leptospirosis suspicious patients were tested with five RDTs, including Medical Science Public Health, Leptocheck-WB, SD bioline, TRUSTline, and J.Mitra. The case definition was based on MAT, qPCR, and culture results. Diagnostic accuracy was determined based on the first day of enrollment in an overall analysis and stratified according to days post-onset of fever. The five RDTs had overall sensitivity ranging from 1.8% to 75% and specificity ranging from 52.3% to 97.7%. Leptocheck-WB had high sensitivity of 75.0%. The sensitivity of five RDTs increased on days 4–6 post-onset of fever, while the specificity of all tests remained relatively stable at different days post-onset of fever.

**Conclusions/Significance:**

The tested RDTs showed low sensitivity. Therefore, based on the present study, five commercially available RDTs might not be an appropriate test for acute leptospirosis screening in the Thai population.

## Author summary

Leptospirosis is an important zoonosis, especially in the tropics. Rapid diagnostic tests (RDTs) have become widely used in low-resource settings for leptospirosis diagnostics. In this study, we evaluated the diagnostic performance of five commercially available RDTs in Thai population. The overall sensitivity ranged from 1.8% to 75%, and specificity was 52.3% to 97.7%. Leptocheck-WB had the highest sensitivity of 75.0%. The sensitivity of five RDTs increased on days 4–6 post onset of fever, while the specificity of all tests remained relatively stable at different days post onset of fever. Based on these results, RDTs showed low sensitivity and might not be an appropriate test for acute leptospirosis screening in the Thai population.

## Introduction

Leptospirosis is a globally widespread zoonosis infectious disease caused by *Leptospira* spp. [1,2]. From the reports of the Bureau of Epidemiology, Department of Disease Control, Ministry of Public Health, Thailand, there are more than 2,000 leptospirosis patients each year in Thailand, causing a morbidity rate of approximately 4 per 100,000 population and mortality rate of approximately 0.1 per 100,000 population [3]. Severe leptospirosis may cause multiorgan failure with a high mortality rate [4–6]. The clinical manifestations of leptospirosis, including fever and gastrointestinal symptoms, are non-specific and often challenging to differentiate from other diseases [7].

Rapid diagnosis and management are the keys to reducing mortality in leptospirosis patients. Currently, there are many commercial leptospirosis diagnostic tests available in the market, including Microscopic Agglutination Test (MAT), Enzyme-Linked Immunosorbent Assay (ELISA), Immune Fluorescence Assay (IFA) and Polymerase Chain Reaction (PCR). The purpose of these tests are to detect either leptospirosis antigens or anti-leptospirosis antibodies using different techniques [8–9]. However, the majority of leptospirosis patients are admitted to small primary hospitals in rural areas where these tests are often unavailable.

Rapid diagnostic tests (RDTs) are helpful solutions to minimize the requirement of an advanced laboratory tests in these local settings. In Thailand, there are five rapid diagnostic tests available for leptospirosis IgM. There is no study comparing the performance of these tests in the Thai population. In this study, we aimed to compare the accuracy of the five rapid test brand currently used in Thailand, including “Medical Science Public Health” (Department of Medical Sciences Ministry of Public Health, Thailand), “Leptocheck-WB” (Zephyr Biomedicals, India), “SD bioline” (Standard Diagnostics, South Korea), “TRUSTline” (Athenese-Dx, India) and “J.Mitra” (J.Mitra, India). Diagnostic accuracy was determined based on the first day of enrollment in an overall analysis and stratified according to days post-onset of fever.

## Materials and methods

### Ethics statement

The study protocol was approved by the Central Research Ethics Committee (COA-CREC 005/2017). All participants gave written informed consent, and the study was conducted according to the Helsinki Declaration and Good Clinical Practice guidelines.

### Patients and study design

We evaluated the performance of the five RDT kits among participants with known leptospirosis status (infected or non-infected) from previous studies carried out in Srisaket province, located in eastern Thailand. Subjects were recruited between December 2015—November 2016 from fifteen hospitals in the Srisaket province. We enrolled all patients suspected of leptospirosis who were admitted participanting hospitals. The inclusion and exclusion criteria were determined by a clinician. Specific inclusion criteria included (1) age more than 18 years old, (2) high fever (body temperature higher than 38°C) and (3) history of exposure to reservoir animals or flood water. Exclusion criteria included patients who suffered from other known infectious diseases. The relevance of clinical data was obtained from patient hospital charts. Blood samples were serially collected on the first of enrollment and day 7 after enrollment. Blood samples were then stored at -80°C until analyzed.

### Leptospirosis case definition

We used three standard techniques to confirm leptospirosis, which included microscopic agglutination test (MAT) [10], direct culture in blood, and real-time PCR in blood (qPCR). Leptospirosis suspected cases were classified as Leptospirosis confirmed cases if any one of the above tests was positive. Positive MAT was defined as a single serum titer of ≥ 1:400 or a 4-fold rise in pair serum (7 days apart). For the direct culture of leptospires, 1 mL of whole fresh blood was added into 4 mL of Ellinghausen, McCullough, Johnson and Harris (EMJH) medium [11–13] using a syringe and incubated at 30°C for 2 weeks. Detection for leptospires was accomplished by direct observation using Dark-field microscopy. The qPCR targeting the LipL32 gene was performed as previously described by Stoddard RA et al [14]. The assay was found to be 100% sensitive and specific for pathogenic *Leptospira spp*. DNA. Total DNA was extracted from 200 ul of whole blood samples using a High Pure PCR Template Preparation kit (Roche Diagnostics, Germany). Pre-validated specific primers and TaqMan probe targeting lipL32 from Stoddard RA et al. were used in this study. The qPCR product size was 242 bp. The two primers used were as follows 45F primers (5’ AAG CAT TAC CGC TTG TGG TG3’) and 286R primers (5’ GAA CTC CCA TT T CAG CGA 3’) and Probe 189P (FAM-5’-AA AGC CAG GAC AAG CGC CG-3’-QSY). The qPCR reactions were performed in a final volume of 20 μl, corresponding to 5 μl of genomic DNA and 15 μl of reaction mix containing; the 10 μl SsoAdvanced Universal Probe Supermixs (Biorad Laboratories, USA) part number 64182275, providing final concentrations of 10μM of each primer and 10 μM of the FAM-QSY labelled probe. A no template control (NTC) that contained all the above reagents was also included to detect the presence of contaminating DNA. Amplification and fluorescence detection were conducted in the StepOnePlus Real-Time PCR Systems (Applied Biosystems, USA) with a program of 40 cycles, each cycle consisting of 95°C for 15 seconds and 60°C for one minute. The qPCR reactions were performed in duplicate. A negative result was assigned where no amplification occurred, i.e. the threshold cycle (Ct) value was greater than 40 cycles [15].

### Rapid diagnostic testing procedure

We tested only blood samples from the first day of enrollment to evaluate the early diagnosis accuracy of five RDTs. The 99 blood samples from the first day of enrollment, which included 56 leptospirosis and 43 non-leptospirosis patients, were tested. The kits are designed to detect *Leptospira* IgM antibody. The principle of five RDTs is based on lateral flow chromatographic immunoassay (S1 Table).

The five RDTs were “Medical Science Public Health” (Department of Medical Sciences, Ministry of Public Health, Thailand) defined as RDT1, “Leptocheck-WB” (Zephyr Biomedicals, India) defined as RDT2, “SD bioline” (Standard Diagnostics, South Korea) defined as RDT3, “TRUSTline” (Athenese-Dx, India) defined as RDT4 and “J.Mitra” (J.Mitra, India) defined as RDT5. The RDT test kit components were used according to manufacturer’s instructions. Briefly, the serum specimen and the test components were thawed to room temperature (RT). Then, the test device was labeled with the patient’s identity (patient’s code). Serum specimen (the volume depended on each manufacturer’s instructions) was added into sample well without air bubbles. Then, assay diluent was immediately added to the assay diluent well. The device was kept at RT and the results were read at the end of 15–20 minutes (depended on each manufacturer’s instructions)

Each test was interpreted by three certified laboratory technicians who were trained before the start of the study. Training included classifying a qualitative test outcome as “positive” or “negative” as described by the manufacturer’s instructions. The tests were considered to be positive if at least 2 of 3 technicians read the results as positive.

### Statistical analysis

Continuous variables are presented as the mean ± one standard deviation (SD) in case of a normal distribution, and as a median and interquartile range (IQR) in case of non-normally distributed variables. Student’s t-test or Mann-Whitney test was used to analyze the differences between two continuous variables. Categorical variables were characterized by numbers with percentages and were compared using the Chi-square test. The diagnostic accuracy of RDTs was evaluated. The performance of RDTs was expressed by calculating the sensitivity, specificity, positive and negative predictive values, and the area under the receiver operator characteristics (ROC) curve. To show the change in sensitivity of the tests with time after the onset of fever, we divided patients into three subgroups by the onset of fever (at first day of enrollment), which included within 3 days from fever onset, within 4–6 days from fever onset, and after 7 days from fever onset. Then, the data were analyzed accordingly. All statistical analyses were performed with SPSS Version 22 software (SPSS, Chicago, IL), and figures were drawn using GraphPad Prism 8 (GraphPad Software Inc., California, USA).

## Results

### Study population

**Fig 1:**
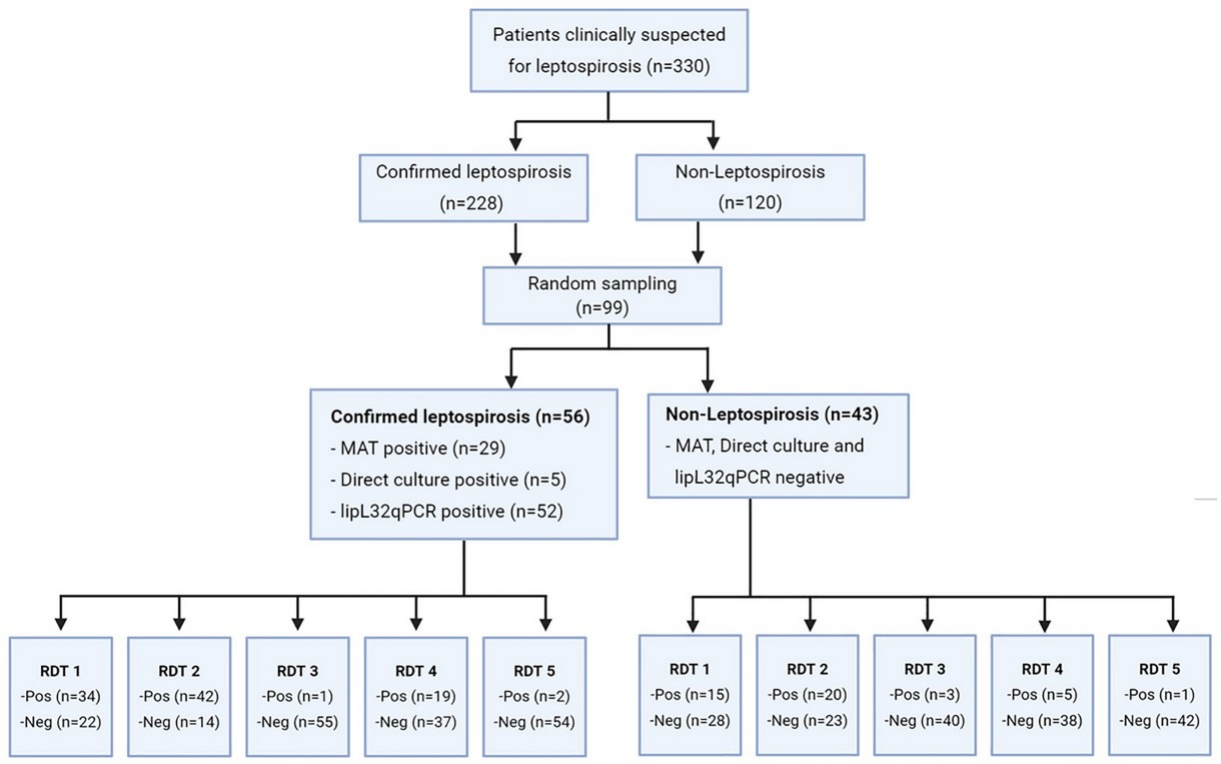
Flowchart of participants.

There were a total of 330 patients fulfilling our inclusion criteria, 228 (69.1%) were leptospirosis cases and 102 (30.9%) were non-leptospirosis cases. We randomly selected 99 stored serum samples for RDT evaluations. Among those, 56 (56.5%) were leptospirosis confirmed cases, and 43 (43.4%) were confirmed not to have leptospirosis. Among 56 confirmed leptospirosis cases, 29 (51.8%) were MAT positive, 5 (8.9%) were culture positive and 52 (92.9%) were real-time PCR positive (Fig 1).

Comparisons of clinical characteristics between leptospirosis and non-leptospirosis groups at the time of enrollment are shown in Table 1. Compared with non-leptospirosis, leptospirosis patients had significantly lower body temperature, serum sodium, serum bicarbonate levels, and platelet count but higher serum creatinine levels. Blood pressure and other relevant laboratory investigations were not significantly different between groups.

**Table 1:** Patients’ clinical and laboratory characteristics of confirmed-leptospirosis and non-leptospirosis groups.

Characteristic	Leptospirosis (N = 56)	Non-leptospirosis (N = 43)	Total (N = 99)	p-value
Male gender, n (%)	42 (75.0%)	35 (81.4%)	77 (77.8%)	0.448
Age, years, Mean (SD)	46.09 (18.21)	48 (15)	46 (17)	0.570
Days of fever until enrollment, Median (IQR)	4 (3, 6)	4 (2, 5)	4 (2, 6)	0.250
Exposure to flood waters, n (%)	41(73.2)	36(83.7)	77(77.0)	0.437
Close contact with animals, n (%)	10(17.9)	7(16.3)	17(17.0)	0.741
Leptospira interogans Serovars				NA
Shermani	20	0	20	
Australis	6	0	6	
Sejroe	4	0	4	
Louisaina	1	0	1	
Grippotyphosa	1	0	1	
Autumnalis	1	0	1	
Semaranga	1	0	1	
Body temperature, Mean (SD)	37.79 (1.08)	38.34 (1.41)	38.03 (1.26)	0.034*
SBP, mmHg, Median (IQR)	110.00 (97.00, 126.75)	113.00 (100.00, 130.00)	112.000 (100.00, 129.00)	0.456
DBP, mmHg, Median (IQR)	66.50 (60.00, 79.00)	68.00 (60.00, 74.00)	68.00 (60.00, 74.00)	0.873
Platelet (x103/uL), Median (IQR)	88500.00 (39000.00, 165250.00)	170000.00 (97500.00, 218000.00)	130000.00 (52000.00, 207500.00)	0.002*
WBC (x103/uL), Mean (SD)	11049.09 (5185.23)	9697.56 (5098.65)	10471.88 (5165.303)	0.206
Creatinine, mg/dL, Median (IQR)	1.28 (1.01, 2.44)	1.00 (0.89, 1.28)	1.12 (0.91, 1.65)	0.003*
TB, g/dL, Median (IQR)	1.18 (0.68, 2.40)	0.75 (0.43, 1.90)	1.00 (0.50, 2.10)	0.055
DB, g/dL, Median (IQR)	0.70 (0.39, 1.60)	0.45 (0.20, 1.05)	0.52 (0.30, 1.16)	0.162
SGOT, U/L, Median (IQR)	60.50 (41.75, 120.00)	53.50 (32.25, 121.00)	56.50 (39.50, 120.00)	0.516
SGPT, U/L, Median (IQR)	48.50 (26.00, 87.75)	45.50 (32.00, 75.75)	46.00 (31.00, 82.00)	0.706
Na, mEq/L, Median (IQR)	135.00 (132.00, 139.00)	139.00 (133.80, 141.00)	137.00 (132.00, 139.00)	0.009*
K, mEq/L, Median (IQR)	3.60 (3.20, 3.90)	3.70 (3.29, 4.00)	3.60 (3.24, 3.90)	0.299
Hco3, mEq/L, Median (IQR)	23.90 (18.85, 25.75)	25.00 (23.50, 26.00)	24.00 (21.00, 26.00)	0.020*

### Overall diagnostic accuracy

**Fig 2:**
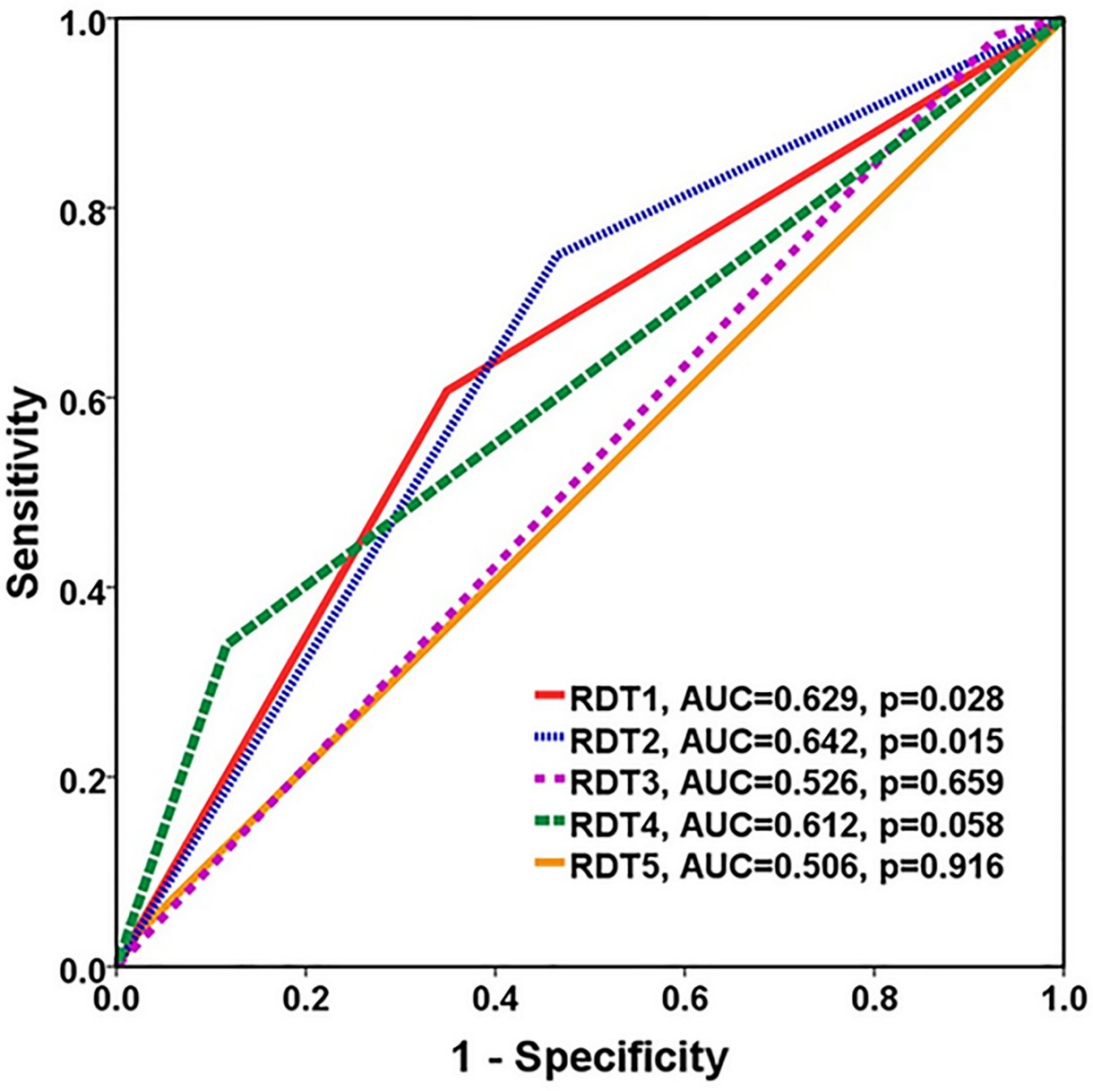
AUC ROC curve comparison against the reference standard method for predicting leptospirosis.

The overall diagnostic accuracy of each RDT were demonstrated in Table 2. Among 56 leptospirosis samples, RDTs showed positive results in 34 (60.70%) for RDT1, 42 (75.0%) for RDT2, 1 (1.8%) for RDT3, 19 (33.9%) for RDT4 and 2 (3.6%) for RDT5. Five RDTs had overall sensitivity ranging from 1.8% to 75% and specificity ranging from 52.3% to 97.7%. The RDT2 and RDT1 had high sensitivity of 75.0% and 60.7%, respectively, while RDT4, RDT5 and RDT3 had low sensitivity of 33.9%, 3.6%, and 1.8%, respectively. On the contrary, RDT5, RDT3 and RDT4 had high specificity 97.7%, 93.0%, and 88.4%, respectively, while RDT2 and RDT1 had only intermediate specificity of 53.5and 65.1, respectively. Each RDT had approximately 60–70% PPV and 40–60% NPV, except RDT3 which had low PPV and NPV of 25% and 42.1%, respectively. Area under the ROC curve for RDTs 1–5 were 0.629, 0.642, 0.526, 0.612 and 0.506, respectively (Fig 2).

**Table 2:** Over all sensitivity, specificity, positive predictive value (PPV) and negative predictive value (NPV) of five rapid diagnostic tests.

Test kit	Sensitivity (%)	Specificity (%)	PPV (%)	NPV (%)
RTD1	60.70	65.10	69.40	56.00
RTD2	75.00	53.50	67.70	62.20
RTD3	1.80	93.00	25.00	42.10
RTD4	33.90	88.40	79.20	50.70
RTD5	3.60	97.70	66.70	43.80

### Diagnostic accuracy at different day post-onset of fever

**Fig 3:**
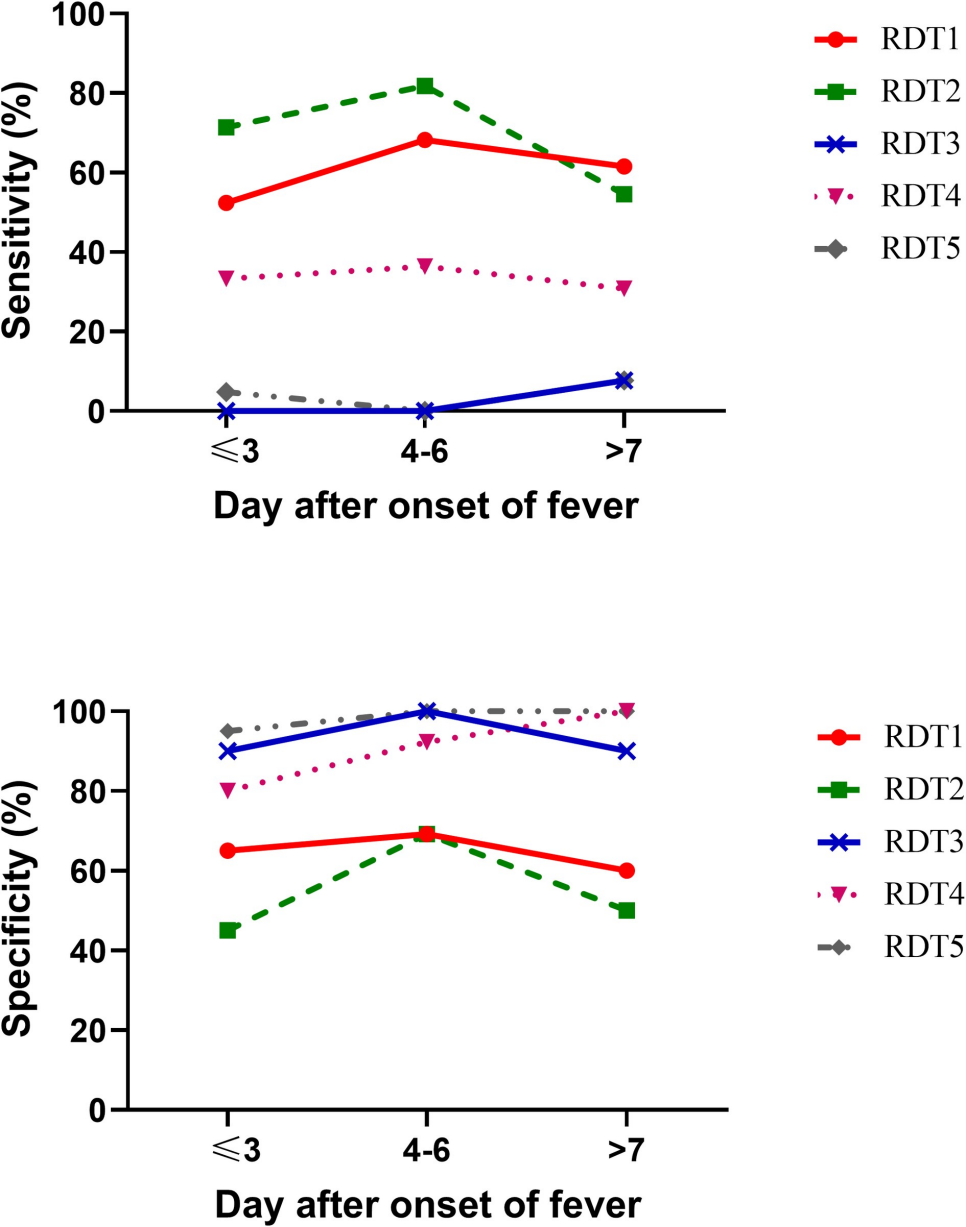
Sensitivity and specificity of five rapid diagnostic tests at different days post onset of fever. (A) The sensitivity (y-axis) relative to day after onset of fever (x-axis). (B) The specificity (y-axis) relative to day after onset of fever (x-axis).

To show the change in sensitivity and specificity of the tests with time after the onset of fever, we stratified patients into three subgroups based on the onset of fever (at first day of enrollment), within 3 days from fever onset (N = 40), within 4–6 days from fever onset (N = 35), and after 7 days from fever onset (N = 24). Overall, the sensitivity of the five RDTs increased on fever days 4–6. In contrast, the sensitivity decreased after day 7 of fever. Results show that RDT2 has the highest sensitivity, both on the first 3 days and within 4–6 days from fever onset, as shown in Fig 3A while the specificity of all tests remained relatively stable, as shown in Fig 3B.

### Interobserver variability

The results of the inter-observer comparison are presented in Table 3. The data shows that the diagnostic variability varied slightly between three readers indicating that readers interpreted results differently. Sensitivity ranged by 3–77% depending on the assay, when different laboratory technicians read the results.

**Table 3:** Inter-observer comparison.

Test kit	Parameter	Reader 1	Reader 2	Reader 3
RDT1	Sensitivity (%)	56.7	56.7	56.7
	Specificity (%)	80.0	80.0	80.0
RDT2	Sensitivity (%)	70.0	76.7	76.7
	Specificity (%)	40.0	35.0	35.0
RDT3	Sensitivity (%)	30.0	26.7	26.7
	Specificity (%)	80.0	90.0	90.0
RDT4	Sensitivity (%)	33.3	33.3	33.3
	Specificity (%)	90.0	90.0	90.0
RDT5	Sensitivity (%)	3.3	3.3	3.3
	Specificity (%)	100.0	100.0	100.0

## Discussion

We reported five RDT accuracies in 56 leptospirosis patients and 43 non-leptospirosis patients. Demographic data were comparable between both groups. Day of fever before enrollment was also not different between the two groups. RDT1 and RDT2 had high sensitivity while RDT3, RDT4, and RDT5 had disappointingly low sensitivity but high specificity. The majority of the test accuracy tended to improve with a long day of fever.

Diagnostic accuracy results varied greatly between studies. Among the five tests, RDT2 showed the highest overall sensitivity and moderate specificity. Data of RDT2 from other studies displayed a broad range of results (sensitivity (55%-98.4%), specificity (73.3%-86.95%), PPV (60.9%-87%) and NPV (73.2%-98.4%)) [7,8,16–19]. Our data indicated that RDT3 had the lowest sensitivity but had high specificity. Our results are consistent with previously published data that also showed low sensitivity (21%) and high specificity (94.8%) [20]. Another study by Panwala et al. presented differing sensitivity of 53.7% and specificity of 60.0% [17]. Compared with data provided in the RDT4 manufacturer leaflet, our data showed significantly lower sensitivity (33.9% vs. 90%) and lower specificity (88.4% vs. 99%). We also found lower sensitivity and specificity of RDT5 compared with manufacturer leaflet data (sensitivity of 3.6% vs. 99.2% and specificity of 97.7% vs. 99.6%). Similar findings were found when comparing our data with the RDT1 manufacturer leaflet. Sensitivity decreased from 100% to 60.7%, and specificity decreased from 97.4% to 65.1%.

Several studies have reported the high sensitivity and specificity of RDTs when MAT was used as a standard reference test [9,17,18,21,22]. The use of different reference standard tests will result in a change of reference population, causing test bias. In our study, we used all three criteria (MAT, qPCR, and culture) recommended by WHO to diagnose the reference leptospirosis population, which is crucial in determining the RDTs accuracy.

We found that among 70 patients with MAT negative, 28 patients were qPCR positive (S1 Fig**)**. Therefore, using only MAT criteria to diagnose leptospirosis, 28 patients with only positive qPCR (MAT negative) will be mistakenly classified as non-leptospirosis cases. Some of the previous studies used only MAT as a standard reference test, while some studies used a combination of qPCR and culture. Serovars of leptospirosis might also effect the RDTs diagnostic performance. The majority of our patients were infected with Shermani and Australis Serovars, which are prevalent in Thailand [23]. There is no definitive explanation for why Serovars affects test accuracy. This may be due to the fact that some Serovars promote antibody production from the host better than others, or some Serovars cause more severe disease, which stimulates more robust humoral immune responses [8]. All of the five RDTs use mechanism of IgM detection, which has two crucial points to be considered. First, IgM cannot be detected in the early stages of infection. According to the kinetics of leptospiral infection in blood, the infection produces leptospiraemia in the first few days after exposure, which is rapidly followed by migration of leptospires to target organs. Anti-Leptospira IgM antibodies are not detectable before 4–5 days after onset of symptoms but appear earlier than IgG and agglutinating antibodies [24,25]. Second, IgM can persist in the blood for years, producing false-positive results [26,27].

We also demonstrated that RDTs accuracy tends to increase with the day of fever (Fig 1). This might be because IgM requires time to rise after the onset of infection. This finding was supported by a previous study from the Netherlands, which found that the sensitivity was increased in the latter day post-onset [8]. Surprisingly, the sensitivity of RDT1, RDT2, and RTD3 was decreased in the day of fever ≥ 7 group compared to the day of fever 4–6 group. This might be due to our limitation that we had only 24 patients in the day of fever ≥ 7 group.

Our study had several strengths. First, we compared test accuracy with the day of fever. Considering that it takes several days to a week for IgM to be detectable, the timing of tests is a crucial factor affecting diagnosis accuracy results. Second, we used all three criteria to diagnose the reference leptospirosis population, which is crucial in determining test accuracy. Third, we collected data prospectively, which allowed us to test all samples with all five RDTs. Finally, we demonstrated interobserver variability between three readers, which underlines that the test should be interpreted cautiously.

Our study was not without limitations. First, we used RDTs to test only serum from the first day of enrollment. Therefore, we could not demonstrate the dynamics of seroconversion in pair serum. However, this reflects real-world practice in which only acute serums are available for testing. Second, because patients usually visit the hospital early after fever onset, we therefore, had a smaller number of patients in the day of fever ≥ 7 group (n = 24), which may affect the accuracy of RDTs. Lastly, this study took place in a single province in Thailand, limiting the generalizability of the results.

In conclusion, all five RDTs failed to produce sufficient diagnostic accuracy for screening or confirmatory tests. The diagnostic accuracy of RDTs depended on the day of fever, target populations, and locations. The result of this study indicates that the clinician should not use RDTs alone for the routine diagnosis. While we still wait for the more accurate test, the clinician should incorporate the exposure history, time of onset, and clinical presentation to guide the early diagnosis of leptospirosis.

## Supporting information

S1 Table Details of five rapid diagnostic tests for the diagnosis of human leptospirosis from manufacturer’s instructions sheet. (DOCX)

S1 Fig Confirmatory diagnosis of leptospirosis based on microscopic agglutination test (MAT) and quantitative polymerase chain reaction (qPCR). (TIF)
