# Supplementary material for: A prospective study to evaluate the accuracy of rapid diagnostic tests for diagnosis of human leptospirosis: Result from THAI-LEPTO AKI study
Source: PLoS Negl Trop Dis. 2021 Feb 19;15(2):e0009159. doi: 10.1371/journal.pntd.0009159 (PMC7894855; doi:10.1371/journal.pntd.0009159)
Supplement: S1 Table — (DOCX) [file pntd.0009159.s001.docx]

**S1 table:** Details of five rapid diagnostic tests for the diagnosis of human leptospirosis from manufacturer's instructions sheet.

| **RDTs Name** | **Principle** | **Specific species** | **Sensitivity (%)** | **Specificity (%)** | **Reference Assay** | **Produced by** |
| --- | --- | --- | --- | --- | --- | --- |
| Medical Science Public Health | Lateral-flow immunochromatography | Undisclosed | 100 | 97.4 | IFA, MAT | Department of Medical Sciences, Ministry of Public Health, Thailand |
| Leptocheck | Lateral-flow immunochromatography | Undisclosed | 90.7 | 93.4 | ELISA | Zephyr Biomedicals, India |
| SD Bioline | Lateral-flow immunochromatography | L.interrogans | 96.7 | 95.8 | ELISA | Standard Diagnostics, Korea |
| Trust Line | Lateral-flow immunochromatography | L.interrogans | 90 | 99 | ELISA | Athenese-Dx, India |
| J.Mitra | Lateral-flow immunochromatography | Undisclosed | 99.22 | 99.61 | ELISA | J.Mitra, India |
