# Supplementary figures and images for: A prospective study to evaluate the accuracy of rapid diagnostic tests for diagnosis of human leptospirosis: Result from THAI-LEPTO AKI study
Source: PLoS Negl Trop Dis. 2021 Feb 19;15(2):e0009159. doi: 10.1371/journal.pntd.0009159 (PMC7894855; doi:10.1371/journal.pntd.0009159)

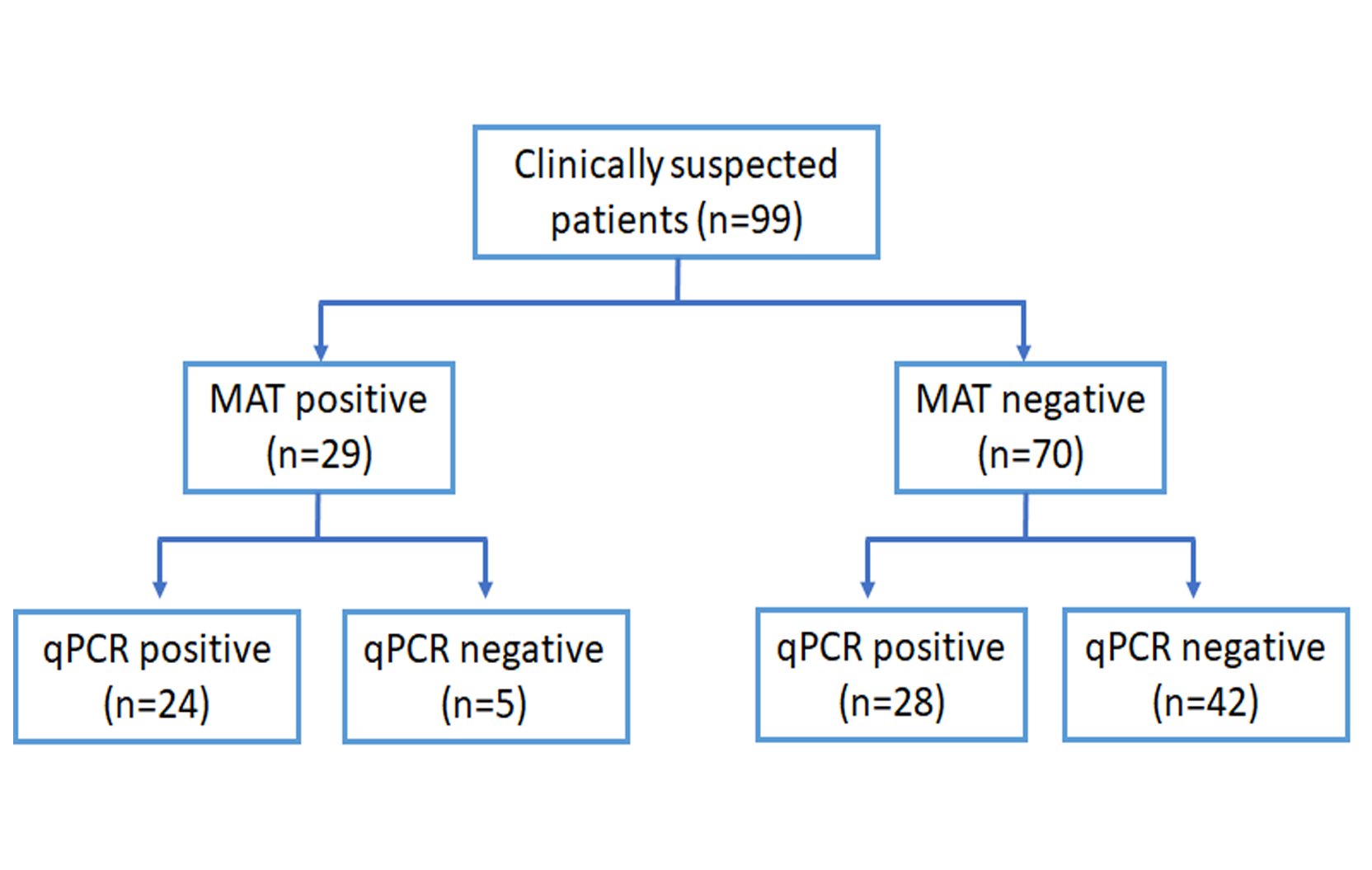

Supplement: S1 Fig — (TIF) [file pntd.0009159.s002.tif]
